# Dysregulated B cell function and disease pathogenesis in systemic sclerosis

**DOI:** 10.3389/fimmu.2022.999008

**Published:** 2023-01-16

**Authors:** Claire F. Beesley, Nina R. Goldman, Taher E. Taher, Christopher P. Denton, David J. Abraham, Rizgar A. Mageed, Voon H. Ong

**Affiliations:** ^1^ Centre for Rheumatology, Division of Medicine, University College London, London, United Kingdom; ^2^ Institute of Immunology and Immunotherapy, University of Birmingham, Birmingham, United Kingdom; ^3^ Centre for Translational Medicine and Therapeutics, William Harvey Research Institute, Queen Mary University of London, London, United Kingdom

**Keywords:** autoantibodies, systemic sclerosis (scleroderma), autoimmunity, B cells, fibrosis

## Abstract

Systemic sclerosis (SSc) is a complex, immune-mediated rheumatic disease characterised by excessive extracellular matrix deposition in the skin and internal organs. B cell infiltration into lesional sites such as the alveolar interstitium and small blood vessels, alongside the production of defined clinically relevant autoantibodies indicates that B cells play a fundamental role in the pathogenesis and development of SSc. This is supported by B cell and fibroblast coculture experiments revealing that B cells directly enhance collagen and extracellular matrix synthesis in fibroblasts. In addition, B cells from SSc patients produce large amounts of profibrotic cytokines such as IL-6 and TGF-β, which interact with other immune and endothelial cells, promoting the profibrotic loop. Furthermore, total B cell counts are increased in SSc patients compared with healthy donors and specific differences can be found in the content of naïve, memory, transitional and regulatory B cell compartments. B cells from SSc patients also show differential expression of activation markers such as CD19 which may shape interactions with other immune mediators such as T follicular helper cells and dendritic cells. The key role of B cells in SSc is further supported by the therapeutic benefit of B cell depletion with rituximab in some patients. It is notable also that B cell signaling is impaired in SSc patients, and this could underpin the failure to induce tolerance in B cells as has been shown in murine models of scleroderma.

## 1 Introduction

Systemic sclerosis (SSc) is a rare, immune-mediated rheumatic disease characterised by pathogenic microvascular damage and progressive fibrosis of the skin and internal organs. It has the highest case-specific mortality of any rheumatic disease and carries significant morbidity for the patient. Although the updated American College of Rheumatology (ACR) and European League Against Rheumatism (EULAR) classification criteria have improved sensitivity and specificity of diagnosis, understanding of disease aetiopathogenesis and development remains unclear (1–3). In addition, SSc is highly heterogenous, so it can be difficult to stratify patients and devise optimal treatment strategies (2, 4).

Skin fibrosis is the major diagnostic feature of SSc, and the extent of skin involvement is used to stratify patients into two subsets. These are limited cutaneous systemic sclerosis (lcSSc) and diffuse cutaneous systemic sclerosis (dcSSc) (5). Patients with lcSSc generally exhibit skin fibrosis which is distal to the elbows and knees, whereas diffuse involvement occurs proximally to the elbows and knees (3). Patients with dcSSc are at an increased risk of complications such as scleroderma renal crisis and interstitial lung disease (ILD), although some patients with lcSSc can also develop these complications (4, 6, 7). Importantly, there is no difference in the frequency and timing of development of significant ILD between patients for both skin subsets (8). Therefore, classification based upon skin subset alone does not offer accurate risk stratification of internal organ involvement.

Pathogenesis of systemic sclerosis is mediated by several immune and inflammatory cells. Currently, it is thought that vascular injury drives an infiltration of mast cells, T lymphocytes and macrophages into lesional tissues early on in the disease. Ultimately, this results in an unresolving pro-inflammatory and pro-fibrotic response mediated by myofibroblast differentiation and the production of cytokines such as interleukin-6 (IL-6) and transforming growth factor β (TGF-β). Evidence for B cell involvement comes from multiple studies outlining dysregulated B cell signaling and homeostasis within SSc patients, as well as evidence from mouse models and B cell modulating therapeutics. The key immunological feature of SSc is the presence of high levels of self-reactive antibodies in the blood. Patients present with various autoantibody profiles and, in clinical practice, this is one of the best indications to stratify patients and predict organ involvement (9). In addition, detection of autoantibodies can precede clinical onset of SSc, highlighting their pathological relevance (10). As such it is likely that autoreactive B cells are a driving factor in SSc, but the complete relevance and origin of these cells has not been fully determined.

## 2 B cell biology

B lymphocytes are pleiotropic cells with multiple functions including antibody and cytokine production, antigen presentation to T cells, modulation of dendritic cell function, and lymphoid organogenesis. As a result, B cells can orchestrate immune responses and influence the local environment at sites of infection and tissue injury/inflammation. With regards to the latter, dysregulated B cell responses have been implicated in a number of autoimmune diseases including systemic lupus erythematosus (SLE), Sjogren’s syndrome and rheumatoid arthritis (RA) (11).

**Figure 1:**
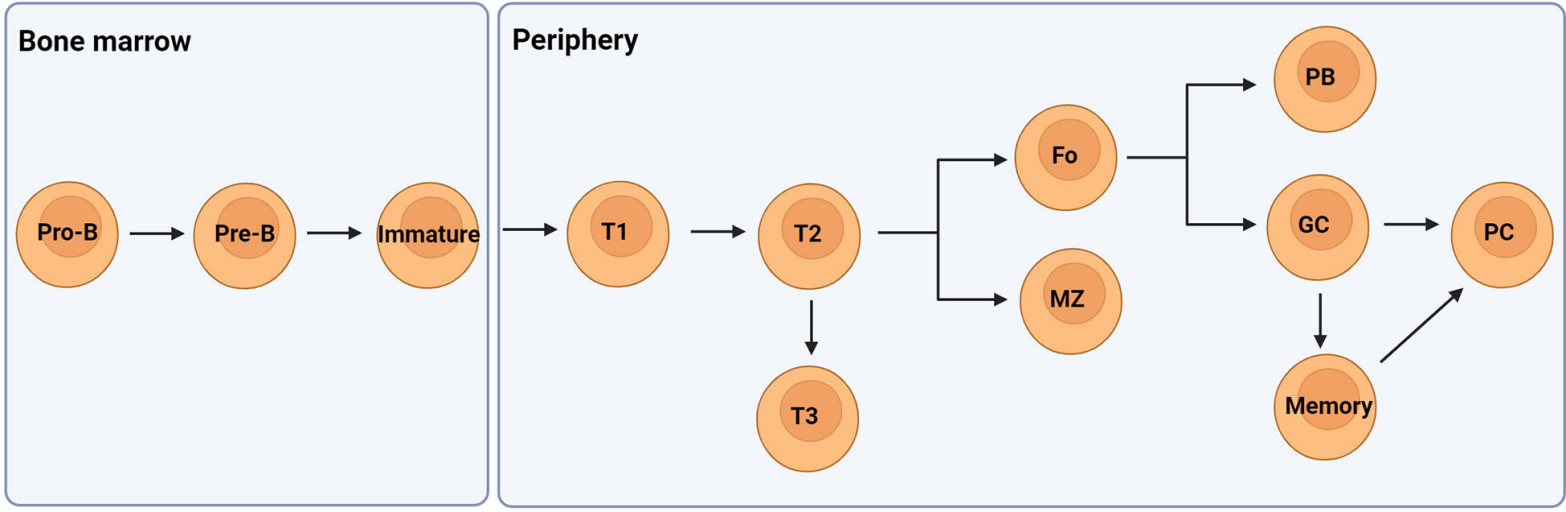
A simplified overview of the developmental pathway of B cells. They develop in the bone marrow from hematopoietic stem cells to pro- and pre-B cells once the IgH genes are rearranged. Following successful IgL gene rearrangement the cells develop to immature B cells expressing IgM. These cells then migrate from the bone marrow to the periphery as transitional 1 (T1) B cells which pass through peripheral checkpoints with most autoreactive B cells becoming anergic-like T3 cells. T2 cells can mature to become either follicular (Fo) or marginal zone (MZ) B cells expressing IgM and IgD. Fo B cells can differentiate to low affinity antibody producing plasmablasts (PB) or undergo class switching and affinity maturation in germinal centres (GC) to become high affinity antibody producing plasma cells or memory cells. Figure created with BioRender.com.

B lymphocytes develop within the bone marrow from hematopoietic stem cells where they acquire a functional B cell receptor (BCR) following rearrangement of their immunoglobulin (Ig) heavy (H) and light (L) genes in the presence of selective growth factors. Following Ig gene rearrangements, immature B cells express surface IgM and are then purged of autoreactive cells that recognise self-antigens in the bone marrow with high affinity. Most self-reactive clones are tolerised at this central checkpoint through mechanisms of receptor editing and clonal deletion. Immature B cell clones that do not recognise local self-antigens leave the bone marrow and migrate to the periphery for development as transitional cells to naïve mature B cells in a linear pathway (**Figure 1**) (12, 13).

## 3 B cell regulation in scleroderma

Differences in B cell homeostasis in SSc patients compared with healthy individuals have been documented in a number of studies (14–16). Typically, these studies have reported an overall increase in the number of B cells and/or differences in the distribution of B cell subsets in scleroderma patients compared with healthy individuals (14–16). However, it is pertinent to highlight that disease duration and immunosuppressive medications are likely to have impacts on lymphocyte homeostasis and subset distribution. Therefore, inclusive analyses are important to mitigate the effect of these factors on altered B cell homeostasis in SSc.

An early study by Sato et al. analysed B cell subsets in 39 Japanese SSc patients who had not received any immunosuppressive therapy. This study observed an increase in the number of circulating CD27^-^ naïve B cells compared with healthy individuals with a reduction in CD27^+^ memory B cells and CD27^+^ plasmablasts (15). Additionally, memory B cells from SSc patients displayed augmented ability to undergo apoptosis and an overexpression of CD19 – possibly relating to their functional hyperactivity (15). These findings have been confirmed in subsequent studies which have revealed further differences between CD27^-^ naïve B cells in healthy individuals and patients. Following from these earlier studies, transitional CD27^-^CD24^hi^CD38^hi^ B cells have also been implicated in scleroderma (17–19). Transitional B cells encompass a heterogenous population comprised of at least three distinct subsets (T1 – T3) with differential expression of IgM and IgD and a differential capacity to respond to antigen stimulation. T1 B cells define a population which has recently emigrated from the bone marrow and must acquire survival signals and undergo peripheral tolerance before the cells can proceed through the T2/3 pathway and develop to maturity (20). The T1 and T2 subsets are distinguishable by their differential capacity to survive or undergo apoptosis following BCR engagement, whilst the T3 subset display a functional status which is similar to that of anergic B cells (20). In some studies, a CD27^+^ subset has also been identified (18, 20). This subset responds rapidly to T cell independent stimulation and is able to produce natural IgM antibodies and secrete high levels of IL-10 (18). Taher et al. studied transitional B cells in SSc patients and reported reduced numbers of T1 cells and expanded T2 cells in SSc patients compared with matched healthy individuals (18). Importantly, T1 cells from SSc patients displayed reduced capacity to undergo apoptosis and contained large numbers of B cells that were specific against the SSc-associated antigen Scl-70. Furthermore, B cell specificity for Scl-70 was demonstrated in patients who were seropositive for Scl-70 autoantibodies, highlighting the significance of this finding (18). This study also analysed the phosphorylation of the STAT-3 signalling pathway when transitional B cells were stimulated through toll-like receptor (TLR)9. STAT-3 has a fundamental role in suppressing pro-inflammatory signal transduction through the TLRs and is crucial for anti-inflammatory IL-10 signalling (21). Taher and colleagues observed a significant reduction in STAT-3 phosphorylation in transitional T1 and CD27^+^ cells from SSc patients and this is consistent with reduced IL-10 production from these cells (18). Taken together this may implicate a failed tolerance checkpoint at the developmental T1 – T2 stage in SSc patients and similar findings have been reported in a recent study by Glauzy and colleagues who concluded that central and peripheral B cell tolerance checkpoints are likely breached in SSc patients (18, 19). However, STAT-3 is also involved in the regulation of several other cytokines including IL-6, indicating that the observation of reduced IL-10 production in SSc B cells may relate to a signalling molecule upstream of STAT-3 (22, 23).

Differences in the number and function of IL-10 producing regulatory B cells, or Bregs have been noted in multiple studies of SSc (24–26). There is no definitive marker to define Bregs, therefore, current classification is based upon Breg capacity to produce high levels of anti-inflammatory IL-10 and immune-regulatory IL-35 and TGF-β – a key cytokine also involved in fibrotic pathways (27). In SSc patients, IL-10 producing B cells are markedly reduced compared with healthy individuals and B cell capacity to produce IL-10 is reduced (16, 18, 24, 25). Diminished Breg populations have been linked with an increased risk of ILD, whilst IL-10 specific B cell expansion has been shown to correlate positively with patient responses to autologous haematopoietic stem cell transplant (16, 25, 28).

A study to examine CD27^+^ memory B cell populations in SSc patients highlighted a reduction in the non-switched memory compartment, resulting in an imbalance between tolerogenic and activated memory B cells (29). In addition, elevated numbers of switched and activated memory B cells were associated with dcSSc and are likely to be relevant through autoantibody and cytokine production. However a significant proportion (52.6%) of the dcSSc patients had received immunosuppressive medications suggesting that these results should be interpreted with caution (29).

## 4 Autoantibodies in SSc

Whilst the pathological relevance of SSc-specific autoantibodies remains incompletely understood, autoantibodies are strongly associated with the disease and are the strongest predictors of disease course and outcome. This means that autoantibodies are valuable clinical tools which are routinely used to stratify patients and predict patient prognosis (9, 30). Typically, these autoantibodies are of high specificity against nuclear antigens and are present at a high concentration (10).

The three autoantibodies which are most frequently associated with SSc are anti-centromere antibodies (ACA), anti-topisomerase I antibodies (ATA) and anti-RNA polymerase III antibodies (ARA). A patient will typically present with one dominant autoantibody specificity and is unlikely to change this autoantibody subtype. One of these three subsets of autoantibodies (ACA, ATA or ARA) are present in over 50% of those with scleroderma (9).

ACA are the most frequently observed autoantibodies in SSc patients (9). These autoantibodies are highly specific for SSc and are associated with lcSSc (31). Most commonly, ACA are specific for centromere protein B (9). Centromere protein B is a highly conserved nuclear protein which facilitates centromere formation. Patients with ACA are often thought to be at risk of developing pulmonary hypertension (PAH) but these studies are either based on enriched cohorts or have used echocardiogram-diagnosed PAH which is not sufficient for PAH diagnosis (32, 33). However, a number of studies have demonstrated that B cell depletion or B cell deficiency can be protective against vascular remodeling in rodent models of PAH (34–36).

ATA positivity is strongly associated with SSc and is present in up to 40% of individuals with the disease. ATA was initially named as anti-Scl-70 as these autoantibodies react with a 70 kDa protein on immunoblots, but it was later realised that Scl-70 was in fact a breakdown product of the larger 100 kDa topoisomerase I protein (37). ATA-positive patients can be dcSSc or lcSSc, although there is a slight predominance of dcSSc with ATA (9). ATA are a strong predictor of digital ulcer, pulmonary fibrosis and ILD development irrespective of skin involvement and ATA levels positively correlate with disease severity and activity (10). Multiple studies have shown that purified ATA from SSc sera can bind to the cell surface of fibroblasts, providing a potential mechanism by which ATA-positivity could influence disease (38, 39).

Unlike anti-RNA polymerase I and II autoantibodies that can be detected across various autoimmune rheumatic diseases, anti-RNA polymerase III antibodies (ARA) are strongly associated with SSc (9). In a recent meta-analysis, the pooled prevalence of ARA positivity in SSc patients was 11% (40). ARA positivity is a strong predictor of scleroderma renal crisis and up to 59% of those with scleroderma renal crisis are seropositive for ARA (41). ARA levels correlate with skin involvement, but they do not predict organ complications or disease outcome (9). However, patients with ARA have a higher risk of cancer (42).

In terms of disease pathogenesis, autoantibodies can amplify immune responses and initiate inflammation and fibrosis through immune complex formation. Recently, a new class of autoantibodies reactive against various cell surface receptors have been identified in those with SSc. These include antibodies with specificity for platelet-derived growth factor receptor alpha (PDGFRα), angiotensin II type 1 receptor (AT1R) and endothelin-1 type A receptor (ETAR). Antibodies against PDGFRα have been linked with fibrosis in both *in vitro* and *in vivo* studies, whilst antibodies against AT1R and ETAR have been linked with vascular damage (43). These autoantibodies, however, are only detected in a small number of SSc patients and have low disease specificity compared with ACA, ATA and ARA positivity (44).

Additionally, autoantibodies with specificity for CD22 have been detected in a subset of patients. CD22 is an inhibitory B cell receptor that dampens BCR signalling *via* a tyrosine phosphorylation-dependent mechanism. Patients with CD22 autoantibodies exhibit significantly worse skin scores than SSc patients without these antibodies and it is hypothesised that anti-CD22 autoantibodies are likely to interfere with CD22-mediated suppression of B cell activation, resulting in further dysregulation of B cell homeostasis (45). However, these autoantibodies have been found in other autoimmune diseases and are only present in a small subset of SSc patients so are not likely to be a driving factor of disease (17, 45).

## 5 Abnormalities in B cell signalling and B cell activation

**Figure 2:**
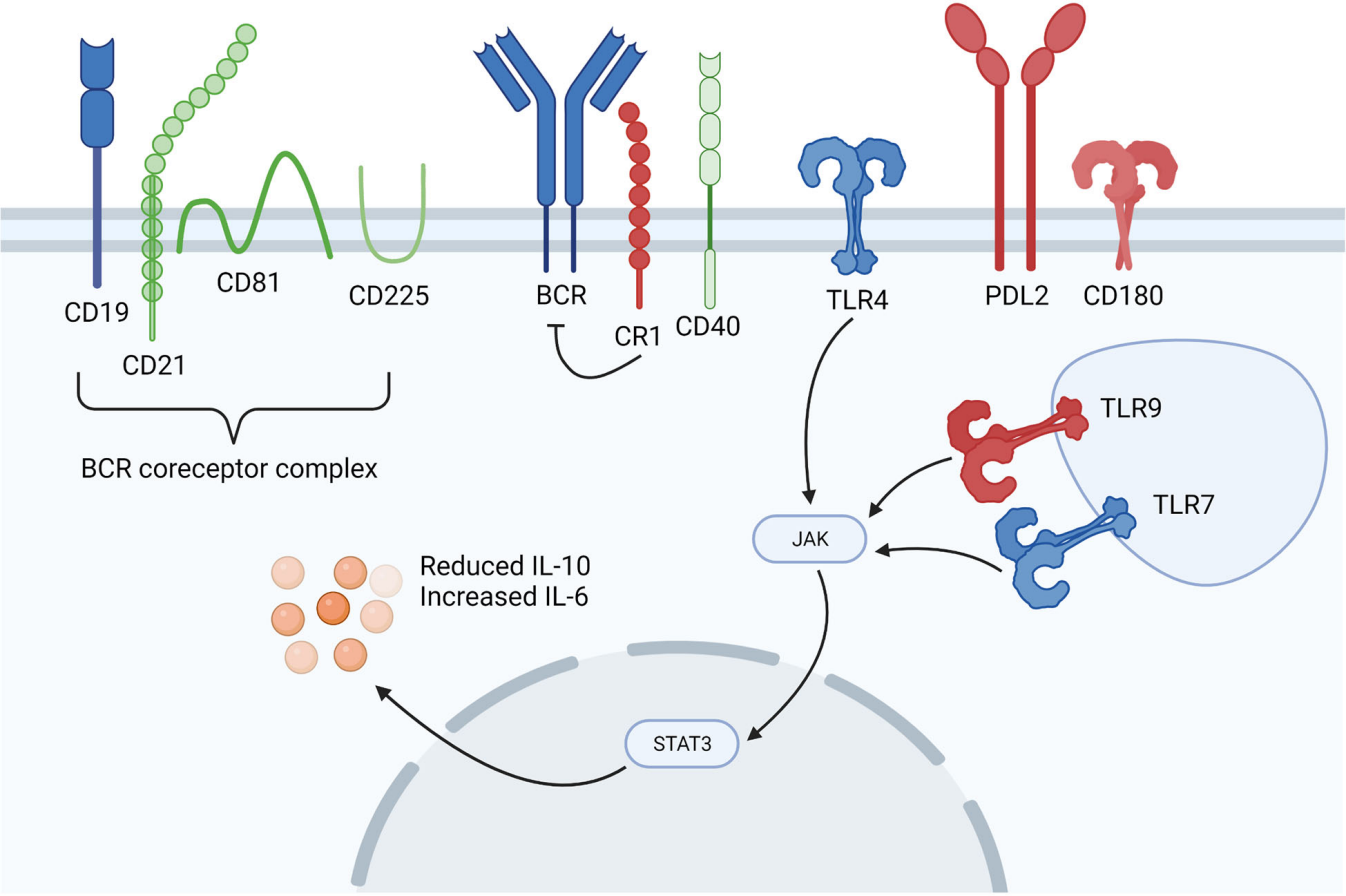
Dysregulated B cell signalling in SSc patients. Overexpressed molecules are shown in blue, underexpressed molecules are shown in red and those shown in green are of interest in SSc, but are neither up or downregulated. SSc B cells are in a hyperactive state marked by differential expression of the BCR coreceptors CD19 and inhibitory CR1. TLR signalling is augmented through differential TLR4, TLR7, TLR9 and CD180 expression, whilst increased levels of serum soluble PD-1 and PD-L2 inhibit the PD-1 and PD-L2 interaction between T and B cells. Figure created with BioRender.com.

In order to mount an effective immune response, B cells must coordinate signalling through multiple innate and adaptive immune receptors. Alongside the BCR, B cells express a variety of coactivating and coinhibitory receptors that regulate B cell activation status. Remarkably, even a minor change in the expression or function of these receptors can result in a defective B cell response. These abnormalities have been reported in murine models of autoimmunity and in patients with autoimmune diseases including SSc (**Figure 2**).

There is considerable evidence to show that B cells in SSc patients are in a hyperactivated state induced, at least partly, by increased levels of BAFF (46). This hyperactivation state is marked by increased expression of the BCR coreceptor CD19 (approximately 20% higher) in SSc patients compared with healthy controls (15, 47). As CD19 lowers the threshold for antigen-dependent stimulation of the BCR, enhanced CD19 expression could augment B cell signalling, resulting in an autoimmune humoral response. Indeed, various autoantibodies, including SSc-specific ATA antibodies were considerably enhanced in transgenic mice that overexpressed CD19 by 20%. This implies that a minor increase in CD19 expression in human SSc may be enough to skew B cell signalling and, thus, trigger autoantibody production (48, 49). CD19 also strengthens antigenic signals generated by the BCR and with CD40 engagement by the CD40 ligand (CD40L) expressed on activated T cells (50). Upregulation of CD19 has been observed in other autoimmune diseases and is considered to be a possible target for future therapy in SSc (17). On B cells, CD19 forms a complex with CD21, also called complement receptor 2 (CR2) which binds to cleavage products of the C3 complement component and transduces signals through CD19, thus lowering the threshold for B cell activation (48). Besides CD21, CD19 also forms a complex with CD225 and CD81, where CD225 could regulate CD19-mediated PI3K signalling (51). On the other hand, CD81 interaction with CD19 is important for CD19 expression and function.

In contrast to CD21, which delivers a costimulatory signal, the complement receptor CD35 (CR1) transduces inhibitory signals by inhibiting the induced increase of cytoplasmic Ca^2+^ levels through BCR and CD40 signalling (17, 52, 53). This suggests that CR1 is a late checkpoint to prevent autoreactive B cell maturation and reduced CR1 expression has been found in memory B cells from SSc patients, potentially augmenting their ability to respond to self-antigen (17).

Programmed cell death protein 1 (PD-1) is a cell surface molecule which is expressed on leukocytes and has been linked to the loss of B cell tolerance and development of autoimmunity. PD-1 regulates immunity by promoting self-tolerance when it is engaged with its ligands PD-L1 and PD-L2 (54, 55). PD-L1 is expressed on many cell types while PD-L2 is expressed on antigen presenting cells. The interaction between PD-1/PD-L2 on B cells and T cells is suggested to suppress tumor necrosis factor alpha (TNF-α) production from antigen-specific B cells and increase IL-10 production (56, 57). This interaction can be blocked by serum soluble PD-1 (sPD-1) and PD-L2 (sPD-L2), resulting in augmented T and B cell responses. Indeed, sPD-1 and sPD-L2 levels are elevated in SSc patients and this correlates with severity of disease, as well as an increase in TNF-α producing B cells and a reduction in IL-10 producing B cells (56, 58).

Signalling through innate immune receptors, such as TLRs can associate with the development of autoimmunity and the loss of B cell tolerance. TLR signalling enables immune cell responses to various stimuli such as pathogen associated molecular signals (PAMPs) and danger associated molecular signals (DAMPs). Defects in TLR signalling have been implicated in several fibrotic diseases and B cell-specific TLR expression dictates developmental trajectories within these cells (59). For example, TLR4 which recognises lipopolysaccharide is thought to promote B cell survival and maturation during transitional development, whilst TLR2 arrests this process (60). In addition, DAMP induced TLR4 activation is known to be a key mediator of myofibroblast differentiation and is relevant in scleroderma as TLR4 and several associated DAMPs are significantly elevated in lesional tissues of SSc patients (61). Additionally, TLR7 and TLR9 can operate in conjunction with BCR-mediated signals and these receptors have important regulatory functions in B cell development and autoimmunity. TLR7 and TLR9 are intracellular endosomal receptors found in eosinophils, dendritic cells and B cells. They recognise bacterial and viral DNA and induce IRF7 signalling and IFN-α production (62). In SLE patients, high TLR7 expression driven by the *TLR7* polymorphism *rs3853839 C/G* was associated with increased disease activity and an upregulation of IFN-responsive genes. Patients with higher TLR7 expression had greater numbers of B cells than patients with lower TLR7 expression and this was most notable in the transitional B cell subset (63). Analysis of the expression of TLR7, TLR9 and JAK2 in PBMC samples from 50 SSc patients and 13 healthy individuals revealed significant TLR7 upregulation in the SSc patients and decreased levels of TLR9 and JAK2. However, this study was carried out using total PBMC and most of the patient cohort were receiving immunosuppressive therapy (64). An *in vitro* study on B cells isolated from SSc patients and healthy donors reported significantly reduced IL-10 production when the SSc B cells were stimulated *via* TLR9 perhaps indicating defective TLR9 signalling in the B cells from SSc patients (18).

Upon BCR engagement, intracellular protein kinases such as SYK and BTK are activated. These kinases then phosphorylate CD19 and B cell adapter for PI3K (BCAP) which provide docking sites for PI3Ks leading to PI3K activation. PI3K activation leads to further downstream activation of Akt and mTOR serine/threonine kinase signalling, as well as NF-κB pathway activation (65–69). These pathways are critical for B cell survival, proliferation and differentiation with defective class 1A PI3K function preventing B cell development beyond the pre-BCR stage (70). Additionally, ubiquitination is a key mechanism for regulating BCR-driven signalling where inappropriate ubiquitination has been associated with autoimmunity. A20 is a widely expressed deubiquitinating and ubiquitin-editing enzyme which restricts NF-κB signalling and protects against TNF-α induced programmed cell death. A20 has been linked with SSc in multiple genome-wide association studies (GWAS) and single nucleotide polymorphism (SNP) analyses which implicate the *Tnfaip3* gene (71, 72). Tavares and colleagues studied this gene using a floxed allele of *Tnfaip3* to generate mice deficient in A20 in B cells (73). The B cells from these mice were hyper-responsive and displayed enhanced NF-κB signalling through CD40 induced signals. The B cells were also resistant to Fas mediated cell death, likely due to increased expression of anti-apoptotic proteins such as Bcl-x produced *via* the NF-κB pathway, potentially providing a mechanism by which autoreactive B cells in genetically susceptible individuals may survive tolerance checkpoints and develop to maturity in SSc (74).

Similar to other autoimmune diseases, SSc development likely results from a combination of genetic and environmental factors with genome-wide association studies (GWAS) identifying a number of polymorphisms associated with SSc. Some of these polymorphisms relate to B cell signalling, but a 2011 association study of B cell gene polymorphisms in a cohort of 900 SSc patients and 1034 heathy individuals did not find evidence of SSc-associated polymorphisms in CD19, CD20, CD22 and CD24 (68). However, there is evidence for increased SSc susceptibility resulting from genetic polymorphisms in coding domains of other B cell signalling molecules. These include polymorphisms in BANK1, BLK, PTPN22 and CSK (69, 75–77). Some of these polymorphisms are known to be associated with multiple autoimmune diseases and are associated with a patient’s ethnicity and autoantibody subset. For example, the *PTPN22* 1858T risk allele is associated with patients who are seropositive for ATA autoantibodies and results in a memory B cell deficit with reduced responsiveness to antigen stimulation *via* the BCR (77). In addition, polymorphisms in the negative regulators of B and T cell activation, suppressors of cytokine signalling 2 (*SOCS2*) and *SOCS3*, were noted to be associated with SSc (78). Further research is needed to understand the functional relevance of these polymorphisms in SSc disease development.

A recent study analysed the expression of PI3K associated molecules in 21 patients with early dcSSc (79). The study identified altered mRNA expression in PI3K associated signalling molecules including TLR homolog CD180, TLR4 and C3 (79). Co-engagement of CD180 and the BCR enhanced NF-κB phosphorylation in dcSSc B cells, but not in healthy controls, whilst activation *via* CD180 increased the percentage of switched memory B cells in dcSSc patients compared with healthy controls (79). Additionally, in 2001, Koarada and colleagues reported that the percentage of SSc patients with CD180-negative B cells was significantly higher than healthy controls, although not as high as those with Sjogren’s syndrome or dermatomyositis (80). This may be significant as ligation of CD180 induces affinity maturation and programs immature (including T1) and mature B cell subsets to become efficient antigen presenting cells to T follicular helper cells (81, 82). Interestingly, another study confirmed these findings and reported reduced CD180 mRNA expression in B cells from dcSSc patients. In lupus CD180-negative B cells have been described as highly activated and CD180 can be internalised after stimulation, indicating that CD180-negative B cells may result from B cell activation *via* CD180. Of note, anti-CD180 stimulation induced natural autoantibody production and significantly increased the concentration of IL-6 in the supernatant of healthy tonsillar B cells providing a mechanism by which CD180 stimulation could increase the number of CD180-negative autoantibody producing B cells in SSc (83).

## 6 Perturbations in the B cell repertoire in SSc patients

Xiadong Shi and colleagues completed a study to analyse the B cell repertoire in SSc patients compared with healthy controls. These investigators reported differential IGHV-J gene usage in SSc patients compared with healthy controls. In addition, they noted that the average CDR3 region was significantly shorter in SSc patients compared with the healthy controls. This is important as the CDR3 region is the most variable region of the BCR and is the prime determinant of antigen specificity (84). Conversely, using immunoglobulin repertoire analyses in new emigrant/transitional B cells in SSc patients and healthy controls, Glauzy and colleagues observed a significantly higher frequency of long IgH CDR3s which are associated with self-reactivity (19). The discrepancy between these studies may reflect the different B cell populations which were sampled as the CDR3 region varies in length between subsets such as naïve and memory B cells (85). Therefore, more research is needed to determine whether the CDR3 region is involved in autoimmunity in SSc.

## 7 Profibrotic and proinflammatory B cell cytokines

**Figure 3:**
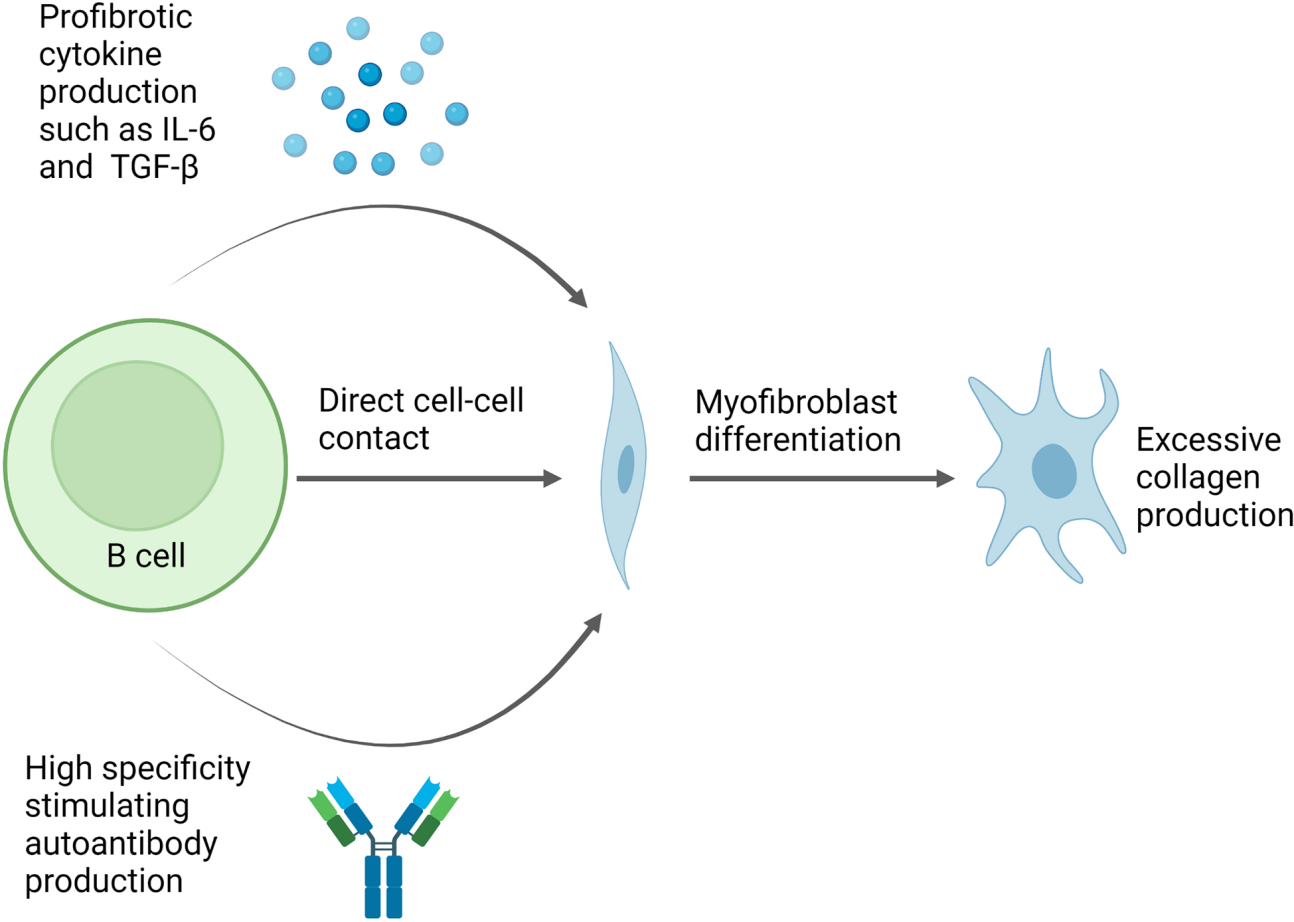
Potential pathway for B cell mediated fibrosis in scleroderma. Direct cell-cell contact can induce changes in gene expression in fibroblasts leading to increased collagen production and myofibroblast differentiation. In addition, B cell production of cytokines such as IL-6 and TGF-β also induce myofibroblast differentiation. Meanwhile, autoantibodies can form immune complexes which bind to fibroblasts and trigger pro-fibrotic effects, whilstsome autoantibody specificities, such as PDGF, observed in a subset of SSc patients can bind fibroblasts directly and have stimulating effects. Figure created with BioRender.com.

B cells contribute to fibrosis *via* a number of mechanisms, including direct cell-cell contact and production of stimulating cytokines such as IL-6 (**Figure 3**). IL-6 is a pleotropic, proinflammatory cytokine which can promote fibrosis (86). Serum levels of IL-6 are increased in SSc patients and this has been linked to worsening disease in human and animal studies (11, 17, 86, 87). IL-6 promotes CD4^+^ T cell differentiation into pro-inflammatory and pro-fibrotic Th1 and Th17 cells (86, 88). Furthermore, B cell derived IL-6 drives spontaneous germinal centre formation in murine lupus, thus, providing a mechanism by which excessive IL-6 production by B cells can result in autoantibody production and autoimmunity (87). IL-6 also acts as a stimulant for B cell proliferation and it enhances plasma cell generation and antibody production, potentially inducing a pathogenic IgG autoantibody response as has been described in a murine model of lupus (89, 90).

Additionally, activated B cells produce high levels of TGF-β which is a central player in fibrosis (91). When TGF-β binds to its receptor on fibroblasts, it induces collagen synthesis and extracellular matrix deposition through Smad signalling (91). In addition, TGF-β promotes fibrosis *via* the inhibition of matrix degrading proteolytic enzymes such as serine proteinases (92). Elevated levels of TGF-β and enhanced expression of its receptors have been found in the skin of SSc patients (93).

IL-10 is a potent anti-inflammatory cytokine and is, therefore, very important in the context of autoimmune disease (94). IL-10 can suppress CD4^+^ T cell proliferation through downregulation of CD86 in an autocrine manner. This also reduces IFN-γ and TNF-α production which are functionally important in scleroderma due to their profibrotic and proinflammatory effects (88). In SSc patients, IL-10 producing B cells are markedly reduced compared with healthy controls and B cell capacity to produce IL-10 when stimulated *via* TLR9 is also reduced (18, 95).

IL-13 is another important Th2-type cytokine with several unique effector functions. IL-13 has been demonstrated to mediate tissue fibrosis in asthma, indicating that it is a key regulator of the extracellular matrix (96). In the context of B cells, IL-13 can upregulate MHC class II expression, induce Ig production, promote IgE class switching and induce B cell proliferation and differentiation (96–98). Serum levels of IL-13 are increased in SSc patients and this correlates with levels of C-reactive protein which is a biomarker of inflammation (99). In addition to IL-13, IL-33 is another important Th2 cytokine which is overexpressed in SSc. This cytokine is constitutively expressed at epithelial barrier sites and its overexpression is of interest since IL-33 can drive tissue fibrosis (100). Importantly, chronic exposure to IL-33 can also promote significant BAFF production *via* neutrophils and dendritic cells resulting in germinal centre formation and an IgG autoantibody response (101).

CXCL-13 is a chemokine that regulates B cell migration through secondary lymphoid tissues and is important for neogenesis of ectopic lymphoid structures in the lungs (102). It is overexpressed in SSc patients and CXCL-13 blood concentrations have been linked with worsening prognosis in patients with idiopathic pulmonary fibrosis (95, 103). It is currently thought that CXCL-13 is produced by monocyte-derived macrophages and that its gene expression is controlled by TNF-α and IL-10 (103, 104).

In addition to these key cytokines, high levels of BAFF have long been noted in SSc patients and are associated with disease progression (46). BAFF is a pleiotropic cytokine (also known as BLyS) which promotes B cell proliferation and is a key regulator of peripheral tolerance. BAFF is also a fundamental survival factor and is involved in multiple cell-fate decisions during B cell development (105–107). BAFF overexpression has been linked with autoimmunity in human and animal studies and BAFF inhibition attenuated skin and lung fibrosis in a mouse model of scleroderma (108). As a homologue of BAFF, elevated levels of a proliferation-inducing ligand (APRIL) have also been reported in SSc patients and have been identified as a marker for pulmonary fibrosis, whilst high BAFF levels indicate severe skin sclerosis (109). A recent study by Glauzy and colleagues, however, did not find elevated BAFF levels in SSc patients compared with healthy controls and hence these authors concluded that BAFF is unlikely to be a driving factor in SSc pathogenesis (19).

Finally, T follicular helper (Tfh) cell production of IL-21 drives autoantibody producing plasma cell differentiation from effector B cells and can promote fibrosis through induction of IL-6, TGF-β and CC chemokine ligand 2 (CCL2) and B cell interaction with fibroblasts. In this respect it is notable that SSc fibroblasts have increased IL-21R expression (110).

## 8 B cell involvement in scleroderma pathogenesis: Evidence from animal models

SSc is a complex disease with high patient-patient heterogeneity. As such, it has been difficult to develop an animal model of SSc which captures all aspects of disease pathogenesis. Generally, these models are murine based and have a fibrotic skin and lung signature induced by either pro-fibrotic agents such as bleomycin, or genetic manipulations leading to pro-fibroblast signalling (111).

The tight-skin mouse (TSK) model of SSc is characterised by skin fibrosis and autoantibody production, as well as an abundance of BAFF and overexpression of CD19 (112–114). Using this model, it was shown that B cell activation is important for fibrosis and autoantibody production as CD19 deficiency decreased skin fibrosis and abrogated autoantibody production in TSK mice (113). Moreover, skin fibrosis and autoantibody production were prevented with the use of a BAFF antagonist (114).

Another mouse model of SSc involves using a subcutaneous injection of bleomycin to induce skin and lung fibrosis as well as autoantibody production. In this setting, bleomycin induces hyaluronan production which activates B cells through TLR4 (115). In the bleomycin model for lung fibrosis, it was demonstrated that CD19 signalling is crucial for B cell infiltration into the lung tissue and is associated with up-regulation of the chemokine CXCR3. Loss of CD19 attenuated inflammation and reduced mortality, while CD19 overexpression increased mortality (115). Moreover, transgenic mice that overexpress CD19 spontaneously produce autoantibodies and lose immunotolerance (16, 116).

Sanges and colleagues developed a new murine model of SSc which was induced through daily intradermal injections of hypochlorous acid (HOCL) (26). The authors found significant B cell infiltration in the skin of HOCL injected mice in the later stages of disease but not in the earlier stages when compared with PBS-treated mice. In addition, splenic B cells in HOCL-treated mice produced significantly more IL-6 and CCL3 while IL-10 production was significantly reduced during the early stages of the disease, but levels of IL-10 production matched those of control mice at the later stage (26). This model showed high concordance with observations of B cell perturbations in human scleroderma patients. This is defined by B cell involvement through an early expansion of transitional B cells and late expansion of the mature naïve subset with an overall decrease in the number of Bregs, plasmablasts and memory B cells (26).

Interestingly, a study using a bleomycin-induced model of scleroderma highlighted how B cell-specific IL-6 deficient mice had attenuated skin and lung fibrosis whilst B cell-specific IL-10-deficient mice had more severe fibrosis. Using this model it was also shown that IL-6 producing B effector cells or Beffs infiltrated inflamed skin and were induced to proliferate through BAFF. However, BAFF suppressed Breg generation. In addition, a BAFF antagonist attenuated skin and lung fibrosis and reduced Beffs but not Bregs. These data provide further evidence for a pathogenic role for BAFF and IL-6 produced by B cells in scleroderma models and a protective role for IL-10 (108).

Recently, a new mouse model of SSc has emerged. This model depends on topoisomerase I injection with complete Freud’s adjuvant. The mice then develop skin and lung fibrosis with a defined Th2/Th17 response and increased IL-6 production. Using this model, it was shown that loss of IL-6 expression significantly improved skin and lung fibrosis (117).

## 9 B cell infiltration in the skin and lungs

Various studies have examined the cellular infiltrates in lesional tissue of SSc patients (118–122). Bosello and colleagues characterised the inflammatory cell infiltrate in scleroderma skin and found CD20^+^ B cells in 60.7% of SSc patients. There was an increased number of CD20^+^ B cells in patients with early disease and B cell infiltration appeared to correlate with worsening skin score. Importantly, no CD20^+^ B cells were found in the skin of healthy individuals (120). Evidence suggests that IL-10 producing B cells migrate from the peritoneum to inflamed skin sites where they play important regulatory and protective functions. Hence, if B cell capacity to produce IL-10 is reduced in SSc patients, then the function of these regulatory skin-homing B cells are likely to be impaired (123).

B cell aggregates have been found in lung tissues of SSc patients with ILD, whilst transcriptomic data has also found evidence of lesional B cell infiltration (118, 121, 122). Furthermore, foci of B cell aggregates have been found in lung alveolar interstitium of SSc patients with ILD (122). Recent data using next-generation RNA sequencing in patients with early dcSSc demonstrated B cell signatures in 67% of patients, higher than published data in established disease (121).

## 10 Therapeutic strategies targeting B cells in SSc

The benefits of immunosuppressive therapies for scleroderma lung and skin disease provide further evidence for the role of B cell autoimmunity in SSc. However, the mixed effects of these biologic treatments also reveal the complexity of SSc pathogenesis and the strategic challenges faced when treating this disease (124).

The Scleroderma Lung Study (SLS) 1, comparing oral cyclophosphamide for 1 year vs placebo, reported a small improvement in forced vital capacity (FVC) with the treatment alongside improvements in dyspnoea scores and skin thickening (125). Using single cell analysis, an individual’s B cell profile has been associated with cyclophosphamide response. Of note, cyclophosphamide responders had increased IL-10 producing regulatory B cells and reduced IL-6 effector B cells post-treatment (126). The SLS 2 study subsequently showed equivalence between 24 months of mycophenolate mofetil (MMF) and 12-months of oral cyclophosphamide followed by placebo with the average improvement in FVC of 7-8% (127). *Post-hoc* analysis of both the SENSCIS trial assessing nintedanib (a tyrosine kinase inhibitor) and the RESOLVE-1 trial assessing lenabasum (a cannabinoid type 2 receptor) in SSc, further demonstrated the efficacy of MMF in SSc. MMF inhibits both B and T cell proliferation and antibody production (128–130). With cyclophosphamide and MMF both impacting B cells alongside other mechanisms of action, further research is needed to disentangle the relative importance of their effects on B cell mediated pathogenesis.

Autologous stem cell transplant (aSCT) has emerged as a treatment option for autoimmune diseases with the rationale that a subsequent new self-tolerant immune system develops post-transplant (131). Three randomised controlled trials have supported this approach in patients with severe SSc with the ASSIST and ASTIS studies performing non-myeloablative aSCT and SCOT trial using myelo-ablative aSCT (132–134). All three trials reported sustained improvements in both skin and lung disease compared with standard of care with cyclophosphamide. The SCOT trial reported a lower treatment related mortality than previous studies, however, early treatment mortality due to increased rates of infection remains a concern (133). Gernert and colleagues demonstrated that the predominant B cell population post-transplantation was naïve B cells (CD27^-^/IgD^+^) with reduced percentages of memory B cells at 1-year post aSCT. An increased regulatory B cell phenotype with increased B cell IL-10 production was also found post-aSCT (28). A further study on 22 patients pre- and post- aSCT for SSc demonstrated similar changes in B cell populations with increased naïve B cells over prolonged follow-ups and sustained decreases in unswitched, switched and double negative B cells (135).

Rituximab, a chimeric monoclonal antibody against human CD20 on B cells provides compelling and more specific evidence for the role of B cells in SSc. Initial studies including small case series and open label trials demonstrated promising results with improvements of lung and skin fibrosis with rituximab (136–144). Alongside these studies, an initial observational EUSTAR study comparing 63 patients who received rituximab in routine clinical practice to matched controls found improved skin thickening and stabilisation of ILD with rituximab treatment (145). These findings are supported by mechanistic evidence that dermal B cells are completely, or nearly completely, depleted by rituximab therapy and a significant reduction in IL-6 at 6 months post treatment occurred with IL-6 known to be predictive of decline in FVC (136, 139, 146). A downregulation of fibroblast type I collagen gene expression with rituximab has also been demonstrated and early depletion of peripheral B cells at 2 weeks after rituximab therapy was negatively correlated with % forced vital capacity improvement at week 24 (147, 148).

The subsequent larger prospective EUSTAR study including 254 patients treated with rituximab vs 9575 propensity-score matched patients showed a significant improvement in skin fibrosis but failed to show an effect of rituximab on FVC or carbon monoxide diffusion capacity (DLCO) decline (149). A recent meta-analysis of rituximab in SSc-ILD from Goswami and colleagues, included 20 studies, with 575 SSc patients receiving rituximab treatment. Rituximab improved FVC and DLCO by 4.48% and 3.47%, respectively at 6 months and 7.03% and 4.08% at 12 months. Additionally, there were concomitant reductions in the Modified Rodnan skin score (mRSS) which is used to evaluate skin thickness, with a higher score indicating thicker skin (150). It must be noted that only 2 of the 20 studies included were randomised control trials and neither were double-blinded. Another meta-analysis published in 2020 by Tang and colleagues including 14 studies with 597 participants found rituximab resulted in stability but not improvement of FVC (151).

The DESIRES trial, a double-blind placebo-controlled trial published in 2021, randomised both diffuse and limited SSc patients with an mRSS of ≥10 to receive weekly rituximab for 4 weeks, or placebo with absolute change in mRSS at 6 months as the primary endpoint (152). An improvement in skin score with rituximab vs placebo (absolute change in mRSS at 6 months -6.30 rituximab vs 2.14 placebo, difference -8.44 p<0.0001) was found and although most patients included in this study had relatively mild associated interstitial lung disease (ILD), rituximab did improve % FVC at 24 weeks (0.09% vs -2.87%, difference 2.96%, 95% CI 0.08 – 5.84, p=0.044). This provides more definitive evidence for the role of B cell depletion. We will wait to see if these results are reinforced by the soon to be published randomised controlled RECITAL trial assessing the effect of rituximab vs cyclophosphamide in connective tissue-disease-associated ILD (153).

Changes in autoantibody levels have been inconsistently reported following rituximab therapy (154–156) with the inability of rituximab to deplete autoantibody-producing long lived plasma cells seen in SSc (157). CD19 is expressed on a broader range of B cell subsets than CD20, including earlier B cell precursor cells, plasmablasts and some plasma cells (157). Elevated plasma cell gene signatures have been found in SSc compared with healthy controls and correlated with disease activity (158). A phase-I randomised placebo-controlled trial assessing a humanised monoclonal antibody targeting CD19 (MEDI551, inebilizumab) found it effectively depleted B cells and plasma cells and appeared well tolerated in SSc (159). At baseline patients with a high plasma cell gene signature were more likely to respond to inebilizumab than those with low plasma cell gene signatures, thereby supporting a role for plasma cells in disease pathogenesis (158). Targeting CD38, a type II glycoprotein highly expressed on several B cell subsets including short and long lived plasma cells, may provide another approach to target autoantibody production; however, studies are yet to assess anti-CD38 therapy in SSc (160).

Belimumab, a treatment licensed in SLE, binds soluble BAFF/BLyS. A double blind randomised control trial of belimumab vs placebo with background MMF including 20 SSc patients failed to reach statistical significance but demonstrated greater improvement in mRSS with belimumab compared with MMF alone (161). Responders to belimumab had significant changes in gene expression involved in B cell signalling and fibrotic signalling, consistent with the mechanism of action of the drug (161).

Blocking IL-6 signalling using tocilizumab, a humanized monoclonal antibody against the human IL-6 receptor α chain has been assessed in the phase II FaSScinate trial and subsequent phase III FocuSSced trial (162, 163). Although both failed to meet their primary endpoint regarding difference in mean change in baseline mRSS at week 24 and 48, both found that tociluzumab slowed lung function decline in early active disease (FVC decline >10%: 17% placebo vs 5% tocilizumab). The interaction of IL-6 and B cells discussed earlier in this review suggests some treatment effects are likely driven by effects of the drug on B cells; however, further research is required to delineate this (89, 90).

Both the SENSCIS trial and the ongoing Scleroderma lung study III are assessing the effect of combination therapy with oral anti-fibrotics and MMF. It is therefore likely that future treatment approaches will include a combination of immune modulatory and anti-fibrotic therapy with B cells appearing to play a crucial role in the interaction of these pathways (164, 165).

## 11 Future perspectives

B cells are able to shape SSc pathogenesis through a number of mechanisms as described throughout this review. Improved understanding of these mechanisms is needed to demonstrate the overall relevance and description of these pathogenic B cells and outline their involvement in the wider context of SSc such as their interaction with other immune and endothelial cells. A potential pathway for B cell autoreactivity and pathogenic SSc involvement could be triggered by an initial environmental signal such as high levels of IL-6. This could then lead to inappropriate B cell activation and survival marked by inflammatory cytokine and autoantibody production. Autoreactive B cells are likely to interact with other immune cells throughout this process such as fibroblasts, Tfh cells during germinal centre reactions and CD4^+^ T helper cells in antigen presentation. In the future, increased knowledge of these interactions in the context of SSc will improve overall understanding of disease pathways and could lead to new and more targeted therapeutic strategies with less side effects.

Thus, as studies continue to delineate the relevant pathogenic B cell signalling pathways, disease treatment is likely to significantly improve with better and safer biologic therapies. For example, improved understanding of the biomarkers which predict patient response to B cell modulating drugs through single-cell RNA sequencing is likely to improve patient stratification. Meanwhile, other studies may reveal B cell signalling pathways or protein targets that are responsible for maintaining the pro-fibrotic disease state.

## 12 Conclusions

SSc is a complex disease with multiple cell types and pathways likely to contribute towards pathogenesis. B cells can interact with many of these pathways directly contributing to the inflammatory and profibrotic phenotype characteristic of SSc. It is currently thought that the initial stages of disease are marked by a proinflammatory response which later leads to a more pronounced fibrotic response. This has implications for when to treat patients and with which biologics. No matter the disease stage, B cells are key immune mediators and are, therefore, likely to be central in scleroderma pathology. As understanding of disease development, B cell involvement and patient heterogeneity continue to improve, this will result in improved patient prognosis in SSc.

## Author contributions

CB and NG wrote the draft manuscript with VO, TT, DA, CD, and RM as editors. All authors contributed to the text and approved the final submission. CB was responsible for figure design and legend assembly.
